# Phenotypic Diversity and Emerging New Tools to Study Macrophage Activation in Bacterial Infectious Diseases

**DOI:** 10.3389/fimmu.2014.00500

**Published:** 2014-10-10

**Authors:** Mignane B. Ka, Aurélie Daumas, Julien Textoris, Jean-Louis Mege

**Affiliations:** ^1^Unité de Recherche sur les Maladies Infectieuses Tropicales et Emergentes, UMR 63, CNRS 7278, IRD 198, INSERM U1095, Aix-Marseille Université, Marseille, France; ^2^Unité Mixte bioMérieux-HCL, Hôpital Edouard Herriot, Lyon, France

**Keywords:** macrophage, activation, polarization, infectious diseases, bacteria

## Abstract

Macrophage polarization is a concept that has been useful to describe the different features of macrophage activation related to specific functions. Macrophage polarization is responsible for a dichotomic approach (killing vs. repair) of the host response to bacteria; M1-type conditions are protective, whereas M2-type conditions are associated with bacterial persistence. The use of the polarization concept to classify the features of macrophage activation in infected patients using transcriptional and/or molecular data and to provide biomarkers for diagnosis and prognosis has most often been unsuccessful. The confrontation of polarization with different clinical situations in which monocytes/macrophages encounter bacteria obliged us to reappraise this concept. With the exception of M2-type infectious diseases, such as leprosy and Whipple’s disease, most acute (sepsis) or chronic (Q fever, tuberculosis) infectious diseases do not exhibit polarized monocytes/macrophages. This is also the case for commensals that shape the immune response and for probiotics that alter the immune response independent of macrophage polarization. We propose that the type of myeloid cells (monocytes vs. macrophages) and the kinetics of the immune response (early vs. late responses) are critical variables for understanding macrophage activation in human infectious diseases. Explorating the role of these new markers will provide important tools to better understand complex macrophage physiology.

## Introduction

Why a new review about macrophage polarization during bacterial infectious diseases? The initial analysis of macrophage activation, based on *in vitro* experiments and the use of animal models, suggested a dichotomic classification based on the production of canonical molecules associated with a specific function. The production of nitric oxide is associated with the killing of microorganisms or tumor cells and characterizes M1-type macrophage response whereas the expression of arginase (production of ornithine) is associated with the repair and characterizes M2-type macrophage responses (1). The concept of M1/M2 polarization has been largely popularized because macrophage polarization was considered the reflection of Th1 and Th2 polarization of lymphocytes, although the idea that activation by T cells is required for macrophage polarization is likely incorrect (1). As the Th1/Th2 paradigm has progressively been replaced by several functional statuses over the past years, the meaning of a similar dichotomy of macrophage activation is unknown. During the last years, numerous transcriptional and/or molecular markers associated with M1- or M2-type macrophage responses were found but they did not have a clear relationship with macrophage functions, which has been a source of controversies. We feel that these new markers could provide additional important tools to better understand complex macrophage physiology. In addition, recent advances suggest that monocytes readily available in humans are not able to polarize like mature tissue macrophages. As a consequence, the increasing number of publications in which clinical cohorts are investigated with new tools of macrophage investigation allows a global analysis of the cell responses, which results in a more precise overview of the clinical data. It is likely that the concept of M1/M2 macrophages is likely insufficient to describe human infectious diseases. While M2-type infectious diseases such as leprosy and Whipple’s disease represent a clinical exception; most acute (sepsis) or chronic (Q fever, tuberculosis) bacterial diseases do not exhibit polarized monocytes/macrophages. According to the analysis of Thomas Kuhn, the “paradigm” of macrophage polarization applied to human bacterial diseases suffers from abnormalities that could lead to a paradigm shift to a kinetic vision of macrophage activation.

## The Macrophage Polarization Concept

**Figure 1:**
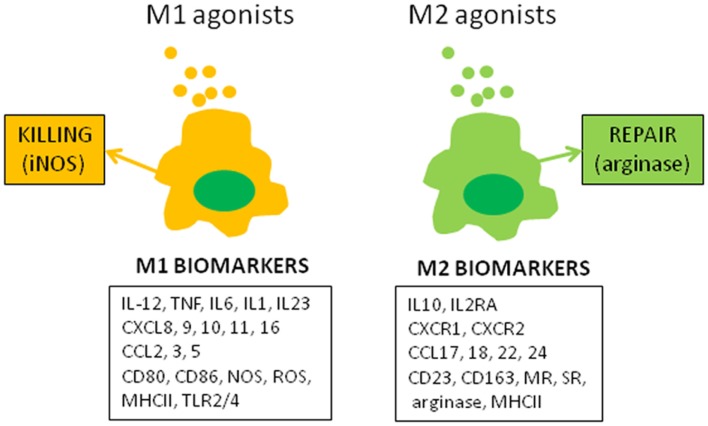
**M1 and M2 macrophage polarization**. The figure represents canonical M1 and M2 agonists that induce the production of M1 and M2 markers by human macrophages *in vitro*. These markers, isolated or combined, have been used to describe the polarization of monocytes and macrophages in clinical investigations.

The molecular concept of the polarization of human macrophages has been initially based on the selective expression of a few markers that have poor specificity when expressed alone. The development of high-throughput profiling technologies that enable the investigation of complex macrophage states (2) has increased the number of biomarkers associated with the M1 or M2 status (Figure 1). Among the papers reporting transcriptomic analysis of activated macrophages that of Martinez et al. was the most contributive (3). The authors showed that M1 and M2 polarization affect 5.2 and 0.3% of transcripts, respectively. The functional annotation reveals the enrichment with categories such as DNA transcription, protein metabolism, G protein coupled-receptors, and lipid metabolism in addition to well-identified cytokine and chemokine families. Hence, the polarization of human macrophages has become more complex than the initial descriptions.

A recent transcriptomic analysis of human macrophages stimulated by a large panel of agonists allowed a description of macrophage activation as a spectrum. This spectrum of activation was more complex than the M1 vs. M2 model of activation because at least nine distinct activation programs were identified. The use of network analyses demonstrated a central transcriptional regulator present in all activation conditions that was complemented by regulators associated with the programs stimulated by each agonist (4). The authors used this model of activation to analyze human alveolar macrophages from patients who were smokers or from patients with chronic obstructive pulmonary disease (COPD). They found that the activation program of macrophages was more complex than predicted in smokers and in patients with COPD. They did not find enrichment with modules associated with interleukin (IL)-4/IL-13 activation in patients with COPD, as was expected, but did find a decrease in the modules associated with interferon (IFN)-γ (4). This report clearly demonstrates that the prominent, popular point of view that cigarette smoke and COPD increase M2-like characteristics (5) was not supported when high-throughput approaches were used.

A proteomics approach has also been used to investigate macrophage polarization. The MALDI-TOF mass spectrometry (MS) technique combined with gel electrophoresis permitted the identification of a large number of soluble or membrane proteins in activated macrophages. This double approach allowed the identification of an M1 signature in human macrophages stimulated with LPS and IFN-γ (6). Recently, we used MALDI-TOF MS to characterize whole eukaryotic cells (7) and the activation status of human macrophages (8). We found that whole-cell MALDI-TOF MS analysis was able to discriminate macrophages according to the type of M1 or M2 agonists and allowed for the identification of different subtypes of M1 or M2 macrophages. The MALDI-TOF MS analysis of pathogen-stimulated macrophages also enabled the detection of pathogen-associated fingerprints that did not correspond to the standard M1/M2 polarization model (8). Taken together, the use of polarization markers other than iNOS and arginase has been controversial. Recently, we proposed guidelines for macrophage activation in which we favored an approach based on a combination of markers instead of isolated canonical markers of polarization (9).

The exploration of tissue macrophages, excepted alveolar macrophages, requires biopsies in infected patients even if it is possible to identify M1 and M2 macrophages in tissues using proteomic or immunohistochemical approaches. Recently, macrophage polarization was investigated in tissues from patients with diseases characterized by a Th1 or Th2 response. M1 macrophages were defined as those expressing CD68 or CD163 with phosphorylated STAT1 (pSTAT1), and M2 macrophages were defined on the basis of the co-expression of CMAF (macrophage activation factor) with CD68 or CD163 (10). The pSTAT1 and CMAF are preferentially associated with M1 and M2 macrophages, respectively. In contrast, CD163, which was considered by several authors as an M2 specific-marker (11), was unable to discriminate M1 and M2 macrophages within pathological tissues. These findings were confirmed by a recent study in which macrophages were differentiated by granulocyte macrophage-colony stimulating factor (GM-CSF) or macrophage-colony stimulating factor (M-CSF) and secondarily polarized by IFN-γ or IL-4/IL-13; CD163 was unable to discriminate the M1 status from the M2 status (12). The investigation of macrophage activation in infected patients concerns essentially circulating monocytes that are accessible after blood collection and purification from blood, but the situation regarding their M1/M2 polarization is complex. Using a microarray approach, we showed that M1/M2 polarization, defined by comparison with the IFN-γ and IL-4 signatures of macrophages, was transient in human monocytes, and gene expression data from published reports showed that not even small signatures of polarized macrophages were found in monocytes (13). Hence, the study of activation in tissue macrophages or circulating monocytes suffers from the lack of convenient tools, suggesting that the concept of macrophage polarization is not convenient. Among the recommendations for reporting macrophage activation, the recommendation precising how macrophages are isolated and which marker combinations are used to measure macrophage activation is likely a solution for the investigation of monocytes *ex vivo* (9).

## Macrophage Polarization and Microbiota

The microorganisms present at the surfaces of mucosa mainly consist of commensals that have developed mutualistic relationships with hosts such as human beings. Indeed, during steady-state conditions, the microbiota influences the efficiency of digestion, controls metabolism, and affects the differentiation and functions of intestinal immune cells, including macrophages. This coevolution has been illustrated by numerous reports based on studies on germ-free animals or antibiotic-treated hosts (14–16). It has been established that the intestinal microbiota maintains a tolerant environment that allows the development of M2-like intestinal macrophages. Indeed, the macrophages from lamina propria show down-regulated expression of innate response receptors and inflammatory functions, but they retain phagocytosis and bactericidal activities (17). It is likely that commensals may directly or indirectly shape the polarization status of intestinal macrophages. Hence, *Bacteroides fragilis* and intestinal *Clostridia* are known to stimulate regulatory T cells (Tregs) and polarization toward an M2 phenotype (14). The exopolysaccharide from *Bacillus subtilis* prevents the intestinal disease associated with *Citrobacter rodentium*, and protection is transferred by peritoneal macrophages (18). The probiotic *Clostridium butyricum* promotes the development of IL-10-producing macrophages that prevent inflammatory colitis (19). Some end-products of bacterial anaerobic fermentation, such as short-chain fatty acids (α-butyrate), inhibit the inflammatory response of macrophages via a mechanism based on the inhibition of histone deacetylase (20). In contrast, intestinal commensals such as *Enterococcus faecalis* polarize colon macrophages to an M1 phenotype in a murine model in which macrophages are depleted with clodronate (21). These findings suggest that the diversity of commensal bacteria accounts for the diversity of macrophage responses. Probiotics such as *Lactobacillus* sp. or *Bifidobacterium* sp. may benefit the host (14), but we ignore their effect on macrophage polarization. The strain G-101 of *Lactobacillus brevis* inhibits the inflammatory response of mice treated by trinitrobenzenesulfonic acid. This anti-inflammatory property is related to the ability of the bacteria to prevent the expression of M1 markers and to favor M2 markers, likely via the production of IL-10 (22). For other authors, probiotics have either no effect on the polarization of RAW 264.7 macrophages as a readout (23), or these bacteria promote an activation profile of the M1-like type in THP-1 cells stimulated with lipopolysaccharide (LPS) (24). It is noteworthy that all of these studies are limited to *in vitro* experiments or animal models, and the extrapolation to human beings must be careful.

If the hypothesis that a breach of intestinal homeostasis is true, the presence of pathogenic bacteria would interfere with the polarization status of intestinal and systemic macrophages. Hence, an M1 profile would be found in patients with acute typhoid fever due to *Salmonella enterica* serovar Typhi, whereas an M2 signature would be observed in convalescent patients. The M2 response does not mean eradication of the pathogen because persistence of the M2 status favors re-infection, relapses, and development of a carrier state (25, 26). On the other hand, there is an increase in M1 and M2 markers in antrum from patients infected with *Helicobacter pylori* and uncomplicated gastritis. The presence of atrophic gastritis is associated with the expression of M1 polarization. It is predictable that shifting macrophage polarization from the M1 to M2 status is protective in chronic *H. pylori* infection. This may be reminiscent of the association of high levels of CCL18, a typical M2 marker, with prolonged survival of patients with gastric carcinoma (26, 27).

Imbalances in gut microbiota have also been associated with systemic diseases such as allergy. Recently, Kim et al. reported the induction of allergen-induced infiltration of inflammatory cells in mice treated with antibiotics. This treatment alters macrophage functions but reorients alveolar macrophages and circulating monocytes toward an M2 phenotype. This latter response is involved in allergic airway inflammation induced by allergens. Antibiotic treatment facilitates fungal overgrowth that exacerbates airway inflammation. The prostaglandin E2 produced by gut fungi is responsible for eosinophil-mediated inflammation and M2 polarization of macrophages (28). If the concept of macrophage polarization is useful for analyzing the host response to intestinal pathogens, there is no clear evidence that it is a convenient tool to measure the response to commensals and probiotics.

## Macrophage Polarization and Acute Infectious Diseases

As sepsis is a consequence of the systemic inflammatory response to infectious aggression, it was tantalizing to consider sepsis as an M1-associated disease (25). Sepsis can also associate a secondary immunodeficiency in which the polarization of macrophages may be altered, as in LPS tolerance. Indeed, LPS-tolerant macrophages express M2 markers, but not M1 markers, and this phenotype can be reversed by IFN-γ (29). It is thought that the evolution of sepsis is characterized by a transition from an initial M1 response to a secondary M2 response. The interaction of macrophages with pathogens accounts for their initial polarization, and the M1-to-M2 transition should rather involve mechanisms of activation control such as suppressors of cytokine signaling (SOCS) proteins; SOCS1 and SOCS2 are associated with M2 macrophages whereas SOCS3 is overexpressed in M1 cells. A high SOCS1/SOCS3 expression ratio might be a biomarker of M2 cells *in vivo* (30). The fact that M2 bias is associated with the resistance of mice does not account for the poor prognosis of patients who exhibit secondary immune deficiency with an M2 phenotype. Indeed, this latter phase, named immune paralysis, is associated with increased susceptibility to nosocomial infections and late lethality (31). In patients with sepsis, the percentage of monocytes expressing CD163 and CD206 is increased. The increase in monocytes expressing M2-like markers has been associated with a lower proportion of IFN-γ-producing T cells or with a higher proportion of Tregs in patients with sepsis. Nevertheless, enrichment with M2-type monocytes has no impact on sepsis prognosis (32). In others reports, the expression of CD163 by monocytes is accurate for discriminating patients with inflammatory presentation from those with sepsis (33), suggesting that CD163 may be a biomarker of prognosis and that the expression of CD163 by monocytes is higher in non-survivors than in survivors (34). Soluble forms of M2-type markers such as CD163 and CD206 are also increased in patients with sepsis, and their high levels are associated with poor prognosis in sepsis. Although membrane and soluble forms of CD163 share the ability to be biomarkers of prognosis in sepsis, circulating CD163 reflecting the polarization of monocytes or their activation independently of M1/M2 polarization tends to be ignored (34, 35). The measurement of monocyte activation is a partial reflection of the altered immune functions in tissues from patients with sepsis and does not assess the diversity of stimuli that they encounter from the initial pathological event. It is probably more pertinent to consider the level of monocyte activation and not the bias toward a polarized status as a biomarker.

## Interference with M1 Polarization in Chronic Infectious Diseases: Q Fever

As intracellular bacteria subvert host microbicidal effectors *in vitro*, we proposed that they have evolved specific strategies to interfere with M1 polarization (25). The example of Q fever is informative as we have assessed the concept of macrophage polarization in *in vitro* experiments, animal models, and patients. Q fever is a zoonosis caused by *Coxiella burnetii*, an intracellular bacterium related to *Legionellae* species, and for which the major targets are monocytes and macrophages. The severity of the infectious disease is chronic evolution with a risk of endocarditis or vascular infection (36).

The circulating monocytes exhibit a pro-inflammatory M1-type response, which is consistent with epidemiological data showing bacterial clearance in most infected patients when they are challenged by *C. burnetii in vitro*. More surprisingly, monocyte-derived macrophages are polarized toward an atypical M2-type in response to bacterial stimulation. This latter effect is characterized by the release of IL-10, transforming growth factor (TGF)-β, and CCL18 and the expression of the mannose receptor (MR) and of arginase-1, but macrophages also express IL-6 and CXCL8, two molecules that are associated with M1 polarization (37). These differences in monocyte/macrophage activation may account for the unexplained differences in bacterial survival: *C. burnetii* are unable to replicate in monocytes but replicate within macrophages (38). Similar findings were found *in vitro* with *Mycobacterium tuberculosis*, which prevents M1 polarization and activates peroxisome proliferator-activated receptor (PPAR)-γ, which is characteristic of macrophage M2 polarization (25, 39).

Nevertheless, we identified IL-10 as the only cytokine able to induce the replication of *C. burnetii* in monocytes and macrophages, suggesting that IL-10-associated M2 polarization is involved in bacterial replication and tissue persistence. The role of IL-10 in the pathogenesis of chronic infection is strengthened by the correlation of the amount of IL-10 and the chronic evolution of Q fever with the restoration of the microbicidal competence of monocytes when IL-10 was neutralized (40, 41). The engulfment of apoptotic cells by monocytes and macrophages is associated with an M2 program induced by IL-10 and favors the intracellular replication of *C. burnetii*. In contrast, treatment of these M2 polarized myeloid cells with IFN-γ and the uptake of necrotic cells suggest that the M1 program is sufficient to clear *C. burnetii* (42). The role of IL-10 is demonstrated in transgenic mice that constitutively overexpress IL-10 in the macrophage compartment and exhibit sustained infection, as in chronic Q fever. Macrophages from IL-10-overexpressing mice are unable to clear *C. burnetii* infection and exhibit an M2-type transcriptional program in which arginase, MR and Yim1/2 are increased and inflammatory markers are down-modulated (43). The infection of mice overexpressing IL-10, which mimics tuberculosis reactivation, reveals features of M2 macrophages, as reported above in *C. burnetii* infection of mice (26).

Concomitantly, we found that mice deficient for vanin-1, a membrane-anchored pantetheinase that controls tissue inflammation, are permissive for *C. burnetii* and exhibit an activation program in macrophages that is skewed toward an IL-10-associated M2 phenotype (44). Hence, IL-10-mediated polarization of macrophages is necessary for *C. burnetii* persistence in tissues.

**Figure 2:**
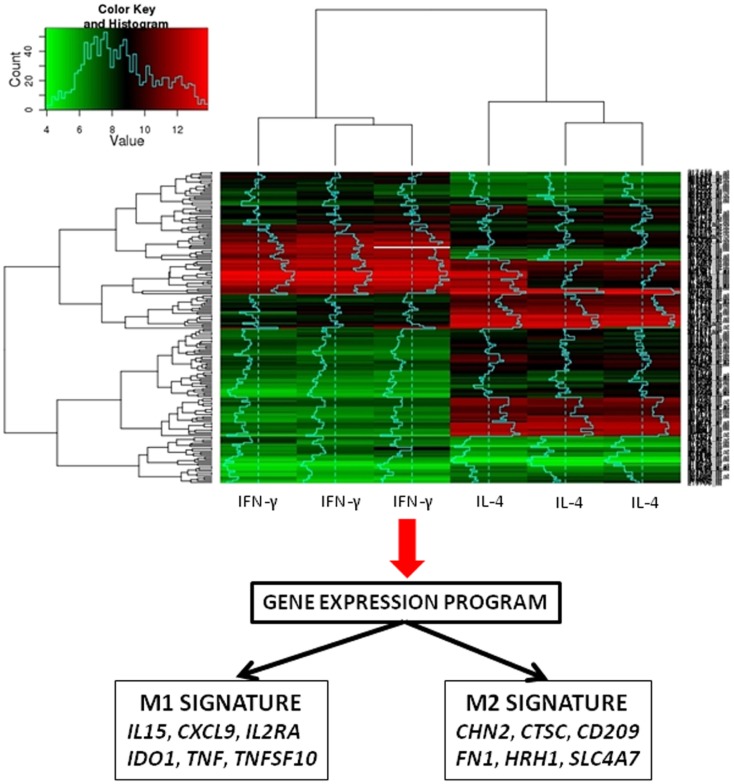
**Transcriptomic assessment of macrophage polarization**. The figure represents the heat map of gene expression in IFN-γ- and IL-4-stimulated macrophages. The use of microarray enables the identification of the original M1 and M2 signatures.

To test the relevance of these findings in patients, we selected M1- and M2-related genes from the microarray analyses of IFN-γ and IL-4-stimulated macrophages (Figure 2). The expression of these genes was not different in patients with acute Q fever and healthy controls. These findings did not support the hypothesis that patients with acute Q fever, who are able to control the infection, should exhibit an M1-type phenotype. The expression of a minority of M1/M2 genes was increased in patients with Q fever endocarditis and who were unable to clear *C. burnetii* and who were expected to exhibit an M2-type phenotype (13). The analysis of the transcriptional profiles of patients with active tuberculosis shows the modulation of M1-related genes, but not that of M2 genes. Similar results were obtained in infants vaccinated with Calmette–Guerin bacillus (26, 45, 46).

In conclusion, the activation program of monocytes from patients with acute and chronic Q fever and tuberculosis cannot be reduced to an M1/M2 dichotomy. We cannot rule out that macrophages in tissues such as endocardium, lungs, or liver are polarized, as suggested by *in vitro* studies and animal models. This is illustrated by the example of pleural macrophages. Tuberculous pleural effusion, an extra-pulmonary form of tuberculosis, is associated with the M1 profile in pleural fluid that is characterized by an increase in M1 macrophages and inflammatory cytokines (47).

## M2 Polarization in Chronic Infectious Diseases: Leprosy and Whipple’s Disease

Two infectious diseases, leprosy and Whipple’s disease, which share several features such as the tropism for macrophages of *Mycobacterium leprae* and *Tropheryma whipplei*, and the role of the immune response into features of pathogenesis, are associated with M2 polarization (26). The overexpression of IL-10 is found in lepromatous lesions and likely reflects M2 polarization. The transcriptional analysis of these lesions reveals an enrichment of M2 genes, which is in contrast to what occurs in tuberculoid lesions (48). The expression of CD163 by foamy macrophages in lepromatous lesions but not by macrophages from tuberculoid lesions has been considered strong evidence of M2 polarization in lepromatous leprosy (26). Whether this polarization is a consequence of the production of IL-10 or if it reflects a Th2 response is often ignored.

Whipple’s disease is characterized by the presence of macrophages with periodic acid-Schiff inclusions within the lamina propria; these macrophages exhibit some features of macrophages from mycobacterial lesions. As described above for lepromatous leprosy, there is converging evidence that Th2 polarization of the immune response is critical for the pathophysiology of Whipple’s disease. An M2 macrophage signature was observed in duodenal biopsies from one patient with intestinal Whipple’s disease (49). Moos et al. reported the increased expression of CD163 on duodenal macrophages and circulating monocytes, and this finding was strengthened by an increase in IL-10 and a decrease in inducible NO synthase expression in these cells, suggesting a functional polarization toward an M2 profile (50, 51). The conclusion that IL-10 may be critical for *T. whipplei* pathogenicity was not confirmed by *in vitro* studies, in which we found an increase in IL-1β, IL-16, and type I IFN production, but not in IL-10 (52, 53). It is likely that type I IFN prevents the IFN-γ-protective effect, as reported for mycobacterial infections (54). This finding underlines the caution that must be taken regarding conclusions about polarization when based on a limited number of markers.

## Complexity of Macrophage Activation in Infectious Diseases

The analysis of infectious disease literature (see above) reveals that modulation of monocyte/macrophage activation is frequently observed, whereas clear-cut M1/M2 polarization is rather a rare event. This observation is related to the history of infected patients. Indeed, the stage of the disease is a critical parameter. For instance, the activation of monocytes/macrophages is different in patients with initial sepsis and those with delayed complications. In addition, numerous patients are distributed between two extreme situations: between patients with acute Q fever and those with Q fever endocarditis, there is a population of patients with valvular disease and Q fever associated with a risk of chronic evolution, and these patients overproduce IL-10 in a sustained manner. However, the measurement of IL-10 at a given time of Q fever evolution is not sufficient to assess the prognosis of patients with Q fever (55). In patients with tuberculosis, the transcriptional signature is transient at the beginning of the disease and is finished 1 year later (45). Clearly, the analysis of the transcriptional pattern of patients with tuberculosis will be dramatically different according to the time of the inclusion, and such an analysis is often difficult to assess at the beginning of the disease. These different clinical and experimental situations drove us to propose a model of monocyte/macrophage activation in which the kinetic component of the disease was integrated. This model is based on the comparison of the transcriptomes from activated monocytes and macrophages. The responses of monocytes to polarizing ligands are characterized by two early and late phases of monocyte activation. The hallmarks of the M1/M2 status are found in the early phase but are absent from the late phase of activation. We selected a series of early and late genes and measured their expression in monocytes from patients with acute and chronic Q fever. Most of the early genes were found to be up-regulated in monocytes from patients with acute Q fever, two of them, NLRC5 and RTP4, were up-regulated by IFN-γ, suggesting that IFN-γ plays a role in the host response during acute Q fever. In contrast, the late genes were up-regulated in chronic Q fever, and some early genes were down-modulated. There was a specific association between late genes such as *ALOX15*, *CLEC4F*, *CCL13*, and *CCL23* and chronic Q fever (13). It is noteworthy that some of them have been associated with the M2 program, which is a result that might lead to incorrect conclusions about monocyte activation. We are unable to assign a function to the modulated genes.

In conclusion, the analysis of macrophage polarization through clinical situations revealed that the mechanisms underlying the activation of monocytes and macrophages are distinct. This point is critical because most clinical investigations are based on monocytes and the conclusions are extrapolated on data obtained with macrophages. The second observation is the importance of activation kinetics in the assessment of infected patients who are at different stages of disease history. Therefore, early and late genes may be alternative biomarkers for analyzing infectious and inflammatory diseases. The lessons from the investigation of infected patients do not invalidate the functional model of M1/M2 polarization. They revealed the difficulty to relate a signature and a function. In addition, the finding of a role for these genes in the activation of macrophages will be useful to understand the complexity of macrophage physiology in normal and pathological conditions.

## Conflict of Interest Statement

The authors declare that the research was conducted in the absence of any commercial or financial relationships that could be construed as a potential conflict of interest.

## References

[1] Mills CD Ley K M1 and M2 macrophages: the chicken and the egg of immunity. J Innate Immun (2014). 10.1159/000364945 25138714 PMC4429858

[2] Kidd BA Peters LA Schadt EE Dudley JT Unifying immunology with informatics and multiscale biology. Nat Immunol (2014) 15(2):118–27 10.1038/ni0914-894c 24448569 PMC4345400

[3] Martinez FO Gordon S Locati M Mantovani A Transcriptional profiling of the human monocyte-to-macrophage differentiation and polarization: new molecules and patterns of gene expression. J Immunol (2006) 177(10):7303–11 10.4049/jimmunol.177.10.7303 17082649

[4] Xue J Schmidt SV Sander J Draffehn A Krebs W Quester I Transcriptome-based network analysis reveals a spectrum model of human macrophage activation. Immunity (2014) 40(2):274–88 10.1016/j.immuni.2014.01.006 24530056 PMC3991396

[5] Hussell T Bell TJ Alveolar macrophages: plasticity in a tissue-specific context. Nat Rev Immunol (2014) 14(2):81–93 10.1038/nri3600 24445666

[6] Brown J Wallet MA Krastins B Sarracino D Goodenow MM Proteome bioprofiles distinguish between M1 priming and activation states in human macrophages. J Leukoc Biol (2010) 87(4):655–62 10.1189/jlb.0809570 20007246 PMC2858305

[7] Ouedraogo R Flaudrops C Ben Amara A Capo C Raoult D Mege JL Global analysis of circulating immune cells by matrix-assisted laser desorption ionization time-of-flight mass spectrometry. PLoS One (2010) 5(10):e13691 10.1371/journal.pone.0013691 21060873 PMC2965159

[8] Ouedraogo R Daumas A Ghigo E Capo C Mege JL Textoris J Whole-cell MALDI-TOF MS: a new tool to assess the multifaceted activation of macrophages. J Proteomics (2012) 75(18):5523–32 10.1016/j.jprot.2012.07.046 22967923

[9] Murray PJ Allen JE Biswas SK Fisher EA Gilroy DW Goerdt S Macrophage activation and polarization: nomenclature and experimental guidelines. Immunity (2014) 41(1):14–20 10.1016/j.immuni.2014.06.008 25035950 PMC4123412

[10] Barros MH Hauck F Dreyer JH Kempkes B Niedobitek G Macrophage polarisation: an immunohistochemical approach for identifying M1 and M2 macrophages. PLoS One (2013) 8(11):e80908 10.1371/journal.pone.0080908 24260507 PMC3829941

[11] Buechler C Ritter M Orso E Langmann T Klucken J Schmitz G Regulation of scavenger receptor CD163 expression in human monocytes and macrophages by pro- and antiinflammatory stimuli. J Leukoc Biol (2000) 67(1):97–103 10.1189/jlb.0813437 10648003

[12] Kittan NA Allen RM Dhaliwal A Cavassani KA Schaller M Gallagher KA Cytokine induced phenotypic and epigenetic signatures are key to establishing specific macrophage phenotypes. PLoS One (2013) 8(10):e78045 10.1371/journal.pone.0078045 24205083 PMC3804553

[13] Mehraj V Textoris J Ben Amara A Ghigo E Raoult D Capo C Monocyte responses in the context of Q fever: from a static polarized model to a kinetic model of activation. J Infect Dis (2013) 208(6):942–51 10.1093/infdis/jit266 23801603

[14] Ivanov II Honda K Intestinal commensal microbes as immune modulators. Cell Host Microbe (2012) 12(4):496–508 10.1016/j.chom.2012.09.009 23084918 PMC3516493

[15] Hooper LV Macpherson AJ Immune adaptations that maintain homeostasis with the intestinal microbiota. Nat Rev Immunol (2010) 10(3):159–69 10.1038/nri2710 20182457

[16] Round JL Mazmanian SK The gut microbiota shapes intestinal immune responses during health and disease. Nat Rev Immunol (2009) 9(5):313–23 10.1038/nri2515 19343057 PMC4095778

[17] Smith PD Ochsenbauer-Jambor C Smythies LE Intestinal macrophages: unique effector cells of the innate immune system. Immunol Rev (2005) 206:149–59 10.1111/j.0105-2896.2005.00288.x 16048547

[18] Jones SE Paynich ML Kearns DB Knight KL Protection from intestinal inflammation by bacterial exopolysaccharides. J Immunol (2014) 192(10):4813–20 10.4049/jimmunol.1303369 24740503 PMC4018721

[19] Hayashi A Sato T Kamada N Mikami Y Matsuoka K Hisamatsu T A single strain of *Clostridium butyricum* induces intestinal IL-10-producing macrophages to suppress acute experimental colitis in mice. Cell Host Microbe (2013) 13(6):711–22 10.1016/j.chom.2013.05.013 23768495

[20] Chang PV Hao L Offermanns S Medzhitov R The microbial metabolite butyrate regulates intestinal macrophage function via histone deacetylase inhibition. Proc Natl Acad Sci U S A (2014) 111(6):2247–52 10.1073/pnas.1322269111 24390544 PMC3926023

[21] Yang Y Wang X Huycke T Moore DR Lightfoot SA Huycke MM Colon macrophages polarized by commensal bacteria cause colitis and cancer through the bystander effect. Transl Oncol (2013) 6(5):596–606 10.1593/tlo.13412 24151540 PMC3799201

[22] Jang SE Hyam SR Han MJ Kim SY Lee BG Kim DH *Lactobacillus brevis* G-101 ameliorates colitis in mice by inhibiting NF-κB, MAPK and AKT pathways and by polarizing M1 macrophages to M2-like macrophages. J Appl Microbiol (2013) 115(3):888–96 10.1111/jam.12273 23742179

[23] Christoffersen TE Hult LT Kuczkowska K Moe KM Skeie S Lea T In vitro comparison of the effects of probiotic, commensal and pathogenic strains on macrophage polarization. Probiotics Antimicrob Proteins (2014) 6(1):1–10 10.1007/s12602-013-9152-0 24676762

[24] Habil N Al-Murrani W Beal J Foey AD Probiotic bacterial strains differentially modulate macrophage cytokine production in a strain-dependent and cell subset-specific manner. Benef Microbes (2011) 2(4):283–93 10.3920/BM2011.0027 22146688

[25] Benoit M Desnues B Mege JL Macrophage polarization in bacterial infections. J Immunol (2008) 181(6):3733–9 10.4049/jimmunol.181.6.3733 18768823

[26] Mege JL Mehraj V Capo C Macrophage polarization and bacterial infections. Curr Opin Infect Dis (2011) 24(3):230–4 10.1097/QCO.0b013e328344b73e 21311324

[27] Quiding-Jarbrink M Raghavan S Sundquist M Enhanced M1 macrophage polarization in human *Helicobacter pylori*-associated atrophic gastritis and in vaccinated mice. PLoS One (2010) 5(11):e15018 10.1371/journal.pone.0015018 21124899 PMC2990716

[28] Kim YG Udayanga KG Totsuka N Weinberg JB Nunez G Shibuya A Gut dysbiosis promotes M2 macrophage polarization and allergic airway inflammation via fungi-induced PGE2. Cell Host Microbe (2014) 15(1):95–102 10.1016/j.chom.2013.12.010 24439901 PMC3957200

[29] Chen J Ivashkiv LB IFN-γ abrogates endotoxin tolerance by facilitating Toll-like receptor-induced chromatin remodeling. Proc Natl Acad Sci U S A (2010) 107(45):19438–43 10.1073/pnas.1007816107 20974955 PMC2984206

[30] Wilson HM SOCS proteins in macrophage polarization and function. Front Immunol (2014) 5:357 10.3389/fimmu.2014.00357 25120543 PMC4112788

[31] Hotchkiss RS Karl IE The pathophysiology and treatment of sepsis. N Engl J Med (2003) 348(2):138–50 10.1056/NEJMra021333 12519925

[32] Brunialti MK Santos MC Rigato O Machado FR Silva E Salomao R Increased percentages of T helper cells producing IL-17 and monocytes expressing markers of alternative activation in patients with sepsis. PLoS One (2012) 7(5):e37393 10.1371/journal.pone.0037393 22693573 PMC3365066

[33] Moller HJ Moestrup SK Weis N Wejse C Nielsen H Pedersen SS Macrophage serum markers in pneumococcal bacteremia: prediction of survival by soluble CD163. Crit Care Med (2006) 34(10):2561–6 10.1097/01.CCM.0000239120.32490.AB 16915112

[34] Kjaergaard AG Rodgaard-Hansen S Dige A Krog J Moller HJ Tonnesen E Monocyte expression and soluble levels of the haemoglobin receptor (CD163/sCD163) and the mannose receptor (MR/sMR) in septic and critically ill non-septic ICU patients. PLoS One (2014) 9(3):e92331 10.1371/journal.pone.0092331 24637679 PMC3956910

[35] Rodgaard-Hansen S Rafique A Christensen PA Maniecki MB Sandahl TD Nexo E A soluble form of the macrophage-related mannose receptor (MR/CD206) is present in human serum and elevated in critical illness. Clin Chem Lab Med (2014) 52(3):453–61 10.1515/cclm-2013-0451 24114918

[36] Raoult D Marrie T Mege JL Natural history and pathophysiology of Q fever. Lancet Infect Dis (2005) 5(4):219–26 10.1016/S1473-3099(05)70052-9 15792739

[37] Benoit M Barbarat B Bernard A Olive D Mege JL *Coxiella burnetii*, the agent of Q fever, stimulates an atypical M2 activation program in human macrophages. Eur J Immunol (2008) 38(4):1065–70 10.1002/eji.200738067 18350541

[38] Ben Amara A Bechah Y Mege JL Immune response and *Coxiella burnetii* invasion. Adv Exp Med Biol (2012) 984:287–98 10.1007/978-94-007-4315-1_15 22711638

[39] Rajaram MV Brooks MN Morris JD Torrelles JB Azad AK Schlesinger LS *Mycobacterium tuberculosis* activates human macrophage peroxisome proliferator-activated receptor gamma linking mannose receptor recognition to regulation of immune responses. J Immunol (2010) 185(2):929–42 10.4049/jimmunol.1000866 20554962 PMC3014549

[40] Ghigo E Capo C Raoult D Mege JL Interleukin-10 stimulates *Coxiella burnetii* replication in human monocytes through tumor necrosis factor down-modulation: role in microbicidal defect of Q fever. Infect Immun (2001) 69(4):2345–52 10.1128/IAI.69.4.2345-2352.2001 11254592 PMC98164

[41] Ghigo E Honstettre A Capo C Gorvel JP Raoult D Mege JL Link between impaired maturation of phagosomes and defective *Coxiella burnetii* killing in patients with chronic Q fever. J Infect Dis (2004) 190(10):1767–72 10.1086/425041 15499532

[42] Benoit M Ghigo E Capo C Raoult D Mege JL The uptake of apoptotic cells drives *Coxiella burnetii* replication and macrophage polarization: a model for Q fever endocarditis. PLoS Pathog (2008) 4(5):e1000066 10.1371/journal.ppat.1000066 18483547 PMC2361190

[43] Meghari S Bechah Y Capo C Lepidi H Raoult D Murray PJ Persistent *Coxiella burnetii* infection in mice overexpressing IL-10: an efficient model for chronic Q fever pathogenesis. PLoS Pathog (2008) 4(2):e23 10.1371/journal.ppat.0040023 18248094 PMC2222951

[44] Meghari S Berruyer C Lepidi H Galland F Naquet P Mege JL Vanin-1 controls granuloma formation and macrophage polarization in Coxiella burnetii infection. Eur J Immunol (2007) 37(1):24–32 10.1002/eji.200636054 17163446

[45] Berry MP Graham CM McNab FW Xu Z Bloch SA Oni T An interferon-inducible neutrophil-driven blood transcriptional signature in human tuberculosis. Nature (2010) 466(7309):973–7 10.1038/nature09247 20725040 PMC3492754

[46] Fletcher HA Keyser A Bowmaker M Sayles PC Kaplan G Hussey G Transcriptional profiling of mycobacterial antigen-induced responses in infants vaccinated with BCG at birth. BMC Med Genomics (2009) 2:10 10.1186/1755-8794-2-10 19239680 PMC2654906

[47] Tang Y Hua SC Qin GX Xu LJ Jiang YF Different subsets of macrophages in patients with new onset tuberculous pleural effusion. PLoS One (2014) 9(2):e88343 10.1371/journal.pone.0088343 24520370 PMC3919770

[48] Montoya D Cruz D Teles RM Lee DJ Ochoa MT Krutzik SR Divergence of macrophage phagocytic and antimicrobial programs in leprosy. Cell Host Microbe (2009) 6(4):343–53 10.1016/j.chom.2009.09.002 19837374 PMC2764558

[49] Desnues B Lepidi H Raoult D Mege JL Whipple disease: intestinal infiltrating cells exhibit a transcriptional pattern of M2/alternatively activated macrophages. J Infect Dis (2005) 192(9):1642–6 10.1086/491745 16206080

[50] Moos V Schmidt C Geelhaar A Kunkel D Allers K Schinnerling K Impaired immune functions of monocytes and macrophages in Whipple’s disease. Gastroenterology (2010) 138(1):210–20 10.1053/j.gastro.2009.07.066 19664628

[51] Geelhaar-Karsch A Schinnerling K Conrad K Friebel J Allers K Schneider T Evaluation of arginine metabolism for the analysis of M1/M2 macrophage activation in human clinical specimens. Inflamm Res (2013) 62(9):865–9 10.1007/s00011-013-0642-z 23775039

[52] Al Moussawi K Ghigo E Kalinke U Alexopoulou L Mege JL Desnues B Type I interferon induction is detrimental during infection with the Whipple’s disease bacterium, *Tropheryma whipplei*. PLoS Pathog (2010) 6(1):e1000722 10.1371/journal.ppat.1000722 20090833 PMC2798751

[53] Desnues B Raoult D Mege JL IL-16 is critical for Tropheryma whipplei replication in Whipple’s disease. J Immunol (2005) 175(7):4575–82 10.4049/jimmunol.175.7.4575 16177102

[54] Rayamajhi M Humann J Kearney S Hill KK Lenz LL Antagonistic crosstalk between type I and II interferons and increased host susceptibility to bacterial infections. Virulence (2010) 1(5):418–22 10.4161/viru.1.5.12787 21178482 PMC2957886

[55] Honstettre A Imbert G Ghigo E Gouriet F Capo C Raoult D Dysregulation of cytokines in acute Q fever: role of interleukin-10 and tumor necrosis factor in chronic evolution of Q fever. J Infect Dis (2003) 187(6):956–62 10.1086/368129 12660942

